# Persons Living with HIV as a Vulnerable Group: An Argument to Ensure Treatment, Care, and Support

**DOI:** 10.3390/tropicalmed9070155

**Published:** 2024-07-11

**Authors:** Michelle Brotherton

**Affiliations:** Philosophy Department, University of Fort Hare, Alice 5700, South Africa; mbrotherton@ufh.ac.za

**Keywords:** vulnerability, HIV status, health status, international law, access to treatment

## Abstract

Generally, human rights documents are to be applied universally. However, certain groups are identified for special treatment due to vulnerabilities faced; these are often referred to as vulnerable groups or populations. While human rights literature and public health literature make a case for particular sensitivity regarding vulnerable populations living with HIV, there is perhaps a case to be made for people living with HIV to be recognised as a vulnerable group in and of itself. It is often other vulnerabilities, such as poverty, disability, or discrimination, that render persons living with HIV legally vulnerable. But what happens if these other vulnerabilities are not present? Persons living with HIV could benefit from being recognised as a vulnerable group, in that it could prioritise their health rights and promote their access to healthcare and services. This article considers how identifying persons living with HIV as a vulnerable group could impact their treatment, care, and support. By looking at examples of countries where people living with HIV have been identified as vulnerable, and at how vulnerable groups are defined, an argument is made that it could be beneficial to persons living with HIV to be identified as a vulnerable group in terms of accessing treatment, care, and support.

## 1. Introduction

“The fight against HIV/AIDS is no longer a battle against the virus, it is, and will increasingly be, a battle for human rights”.

[1]

Imagine a person not being able to receive the necessary treatment, care, and support they need because they are denied a residency permit on the basis of their HIV status [2]. Or imagine someone being denied dental care on the basis of their HIV status and having to incur further costs to receive such care, not because their condition makes it more expensive, but purely because they are being treated in a discriminatory manner on the basis of their HIV status [3]. These are just two examples of instances where a person has not been able to access the care, treatment, or support they need purely because of their HIV status. The reality is that persons living with HIV undergo such discrimination on a daily basis and it affects many facets of their lives, not only as an impediment of access to healthcare. The United Nations Commission on Human Rights has declared that:

“Dscrimination on the basis of AIDS or HIV status, actual or presumed, is prohibited by existing international human rights standards, and that the term ‘or other status’ is non-discrimination provisions in international human rights texts can be interpreted to cover health status, including HIV/AIDS.”

[4]

Non-discrimination clauses in international human rights texts often echo that of the International Covenant on Economic, Social and Cultural Rights (1966), relevant here as we are concerned with health rights and access to healthcare. Article two of the International Covenant on Economic, Social and Cultural Rights states:

“The States Parties to the present Covenant undertake to guarantee that the rights enunciated in the present Covenant will be exercised without discrimination of any kind as to race, colour, sex, language, religion, political or other opinion, national or social origin, property, birth or other status.”

Generally, human rights documents are to be applied universally. However, certain groups are identified for special treatment due to vulnerabilities they face; these are often referred to as vulnerable groups or populations. While human rights texts and public health discourse make a case for particular sensitivity regarding vulnerable populations living with HIV, there is perhaps also a case to be made for people living with HIV to be recognised as a vulnerable group in and of itself. It is often other vulnerabilities, such as poverty, disability, or other kinds of discrimination, that render persons living with HIV legally vulnerable. But what happens if these other vulnerabilities are not present? Persons living with HIV could benefit from being recognised as a vulnerable group, in that it could prioritise their health rights and promote their access to healthcare and services. 

This article considers how recognising persons living with HIV as a vulnerable group could impact their access to treatment, care, and support. In looking at how vulnerable populations are legally defined and what the implications are, and at how non-discrimination clauses lend themselves to providing necessary recognition to persons living with HIV, an argument is made that it could be beneficial to persons living with HIV to be identified as a vulnerable group in terms of realising their rights to health care regarding treatment, care, and support.

## 2. International Human Rights Law, Non-Discrimination, and Vulnerable Groups

International human rights documents are generally universally applied [5]. As Reichert argues:

“After all, considering the universal nature of human rights, why should a particular group be given additional attention? Would this not defeat the purpose of viewing human rights as something for everyone, and not restricted to a special group?”

However, as is often quoted, Aristotle noted that “injustice results as much from treating unequals equally as from treating equals unequally.” [6] Treating everyone the same is not always the fairest approach. As Fineman explains:

“Equality typically is measured by comparing the circumstances of those individuals considered equals. This approach inevitably generates suspicion of unequal or differential treatment absent past discrimination or present stereotyping, particularly if practised by the state.”

[7]

It is for this reason, generally, that human rights documents contain a non-discrimination clause. The purpose of such a clause is to ensure that equity is a possible outcome of treatment and application of rights. As some people are more vulnerable and susceptible to human rights violations than others, differential treatment is justified [8]. According to Reichert, vulnerability “refers to the harsh reality that these groups are more likely to encounter discrimination or other human rights violations than others.” And as Engstrom describes:

“Human life is conditioned by vulnerability. We are all subject to bodily, psychological and socio-political vulnerabilities, as well as to the impact of our natural environment. Vulnerability, in this sense, is an inherent condition of being human in the world, and as much an ontological condition of all human existence.”

[9]

It is because of this inequality in circumstances and conditions that human rights documents make special provision for certain groups. These groups are often defined under the non-discrimination clauses. For example, the Universal Declaration on Human Rights, Article two states:

“Everyone is entitled to all the rights and freedoms set forth in this Declaration, without distinction of any kind, such as race, colour, sex, language, religion, political or other opinion, national or social origin, property, birth or other status.”

[10]

The International Covenant on Civil and Political Rights [11] and the International Covenant on Economic, Social and Cultural Rights [12] echo this clause, as does the African Charter on Human and Peoples Rights [13] and the European Convention on Human Rights [14]. The “or other status” acts as a catch-all phrase, indicating that the list is non-exhaustive and other grounds of discrimination can and do exist. Discrimination is context-dependent [15]. The United Nations Committee on Economic, Social and Cultural Rights issued a General Comment on non-discrimination [16]. The General Comment explicitly states in Article 15 that the “inclusion of ‘other status’ indicates that this list is not exhaustive and other grounds may be incorporated into this category”. The Committee goes on to say (Article 27): “These additional grounds are commonly recognised when they reflect the experience of social groups that are vulnerable and have suffered and continue to suffer marginalization”. 

Health Status is sometimes recognised as a ground of discrimination, falling under the “or other status”. For example, the Constitution of Fiji [17] recognises that a person may not be discriminated against on the grounds of their health status. Health status would inarguably include a person’s HIV status. Recognising health status as a potential ground of discrimination is essential, as Flint argues:

“Given that there is ample evidence and awareness of people experiencing discrimination based on health status, and by not explicitly outlawing discrimination towards people based on health status, implicitly it is accepted. As such, this means that there is a structural process that fosters health inequalities whilst favouring people who are either perceived to be healthy or in some instances, have no visible health decrement or indicator.”

[18]

Discrimination and the stigma that attaches to it can act as a barrier to healthcare services and access to treatment [19]. Stigma and discrimination can even influence population health outcomes “by worsening, undermining, or impeding a number of processes, including social relationships, resource availability, stress, and psychological behavioural responses, exacerbating poor health”. Thus, addressing discrimination is vital for ensuring that adequate healthcare services are provided, and that treatment can be accessed by those in need. Without addressing discrimination and stigma in this regard, perpetual cycles of ill-health and health burdens can occur, leaving those most in need unable to access care because of stigma or discrimination, rendering them further in need of care and treatment.

The International Guidelines on HIV/AIDS and Human Rights, guideline five provides:

“States should enact or strengthen anti-discrimination and other protective laws that protect vulnerable groups, people living with HIV and people living with disabilities from discrimination in both the public and private sectors.”

[20]

Although this does not recognise persons living with HIV as a vulnerable group in and of themselves, it provides the same treatment to persons living with HIV as it does for vulnerable groups, making them comparable and analogous. 

### Recognition of Non-Discrimination on the Basis of HIV Status

Some international documents and some countries have explicitly recognised the right to non-discrimination on the grounds of a person’s HIV status, furthering the argument that persons living with HIV can be considered a vulnerable group under the law, as they are recognised in these examples as deserving of special legal protection. The Constitution of the Republic of Ecuador [21] explicitly recognises that no one shall be discriminated against for health status, or for being an HIV carrier (Article 11(2)). Papua New Guinea has specific legislation in this regard, the HIV/AIDS Management and Prevention Act of 2003, which provides that people may not be discriminated against on the basis of their HIV status and even provides a list of specific things that people cannot be denied on the basis of their HIV status [22]. Pohnei also has legislation in this regard, the HIV Prevention and Care Act of 2007, which provides comprehensive protection against discrimination on the basis of HIV status [23]. Brazil, a leader in human rights-based HIV prevention, treatment, and care [24], have also taken steps to mitigate stigma and discrimination on the basis of HIV status in the Brazilian AIDS Policy [25]. India has approved the HIV/AIDS Prevention Bill [26] and the National Policy on HIV/AIDS and the World of Work [27], which provide non-discrimination on the grounds of a person’s HIV status. South Africa has the Promotion of Equality and Prevention of Unfair Discrimination Act [28], which contains an explicit directive principle for HIV status to be included under prohibitive grounds of discrimination (Article 34). 

Recognition of the right not to be discriminated against on grounds of HIV status, both internationally and in domestic legislation, further reflects the reality that persons living with HIV are subject to discrimination and marginalisation and thereby further entrenches that they are deserving of special protection, like other vulnerable groups. Whilst not all the groups listed under non-discrimination clauses are necessarily considered vulnerable groups, it is recognised that these groups are subject to discrimination more so than others, and thereby one can infer that they are more vulnerable than others, as they are legally entitled to further protection to ensure their rights are protected and realised. 

From these examples of legal and governmental recognition that persons living with HIV should not be discriminated against on the basis of their HIV status, an argument can be made that persons living with HIV can be considered a vulnerable group. It is repeatedly recognised that persons living with HIV are subject to discrimination and marginalisation, otherwise there would not be measures to protect them against such. Furthermore, other social determinants of health play a role in rendering persons vulnerable. Some persons may be more vulnerable than others if subject to compounding vulnerabilities such as low socio-economic status, belonging to an ethnic minority, gender discrimination, religion, or other grounds considered in non-discrimination clauses that could contribute to persons being vulnerable. Compounding factors may leave persons vulnerable beyond living with HIV, and therefore such persons should arguably be prioritised over those not subject to multi-faceted vulnerability, especially when such vulnerabilities may impact determinants of health. Vulnerability can impede the realisation of their rights, such as their right to access to healthcare, affecting their treatment, care, and support. Special provision needs to be made for the more vulnerable to ensure that all persons, regardless of their HIV status, can realise their rights guaranteed in various international human rights law texts and domestic legislation. Despite the universal nature of human rights, special provision is made for the vulnerable. Failing to do so can only result in human rights violations such as the impediment of the realisation of rights. 

## 3. Persons Living with HIV as a Vulnerable Group

The question, then, is whether persons living with HIV can be considered a vulnerable group under non-discrimination clauses, under the “other status” clause where not otherwise explicitly provided for. If persons living with HIV are deserving of special protection under non-discrimination clauses, then it follows that they may be considered a vulnerable group. But what is the purpose of being recognised as a vulnerable group? I argue that there are certain advantages, albeit fair ones, that attach to being recognised as a vulnerable group. As Engstrom argues: “Once individuals or groups can be identified as vulnerable under human rights law, such a recognition implies that there are corresponding duties that follow”. Such duties include special measures to ensure that the rights of persons living with HIV are not violated and that their socio-economic rights such as healthcare are realised and prioritised. 

The discourse on vulnerability is contestable. Fineman in particular raises concerns about how we perceive and treat vulnerability. She proposes a vulnerability theory [29]:

“Rather, addressing human vulnerability calls into focus what we share as human being, what we should expect of the laws and the underlying social structures, and relationships that organize society and affect the lives of everyone within society.”

She explains that laws “are drawn with a created legal subject in mind, an ordinary being, who is the abstract subject of law”. However, her vulnerability theory challenges this limited and arguably inaccurate account of legal subjectivity. She argues for more emphasis to be placed on the shared feature of vulnerability of persons, and that persons are fundamentally defined by their vulnerabilities and needs. However, I am not here to argue for or against a vulnerability theory as pertaining to legal subjectivity. Rather, I argue that it is necessary, within the current constructs of the law, particularly international human rights law, to consider who else may now be considered vulnerable, as this may have changed since these fundamental documents were drafted. Vulnerability is evidently a contemporary consideration in legal theory and the discourse on global justice, as addressing human vulnerability is essential to adequately address the needs of people and society.

So, the question remains: what then, if people living with HIV are recognised as a vulnerable group? What human rights implications are there to recognising that persons living with HIV may be vulnerable as contemplated by non-discrimination clauses in international human rights law documents? The International Guidelines on HIV/AIDS and Human Rights assert that among the human rights principles relevant to HIV/AIDS are “the right to non-discrimination, equal protection and equality before the law”. But what does this mean for persons living with HIV seeking treatment, care, and support? In purporting to answer this question, I will consider a case out of the European Court of Human Rights pertaining to the rights of persons living with HIV, *Kiyutin v. Russia*. 

### 3.1. The Case of Kiyutin v Russia 

*Kiyutin v. Russia* is a landmark case concerning the human rights of persons living with HIV that was heard in the European Court of Human Rights in 2011. The case concerned a Mr Kiyutin, an Uzbek national who was living in Russia with his Russian wife and small child. His application for a residence permit was refused on the basis of his HIV-positive status. Thus, at issue before the court was Article 14 of the European Convention on Human Rights that provides for the prohibition of discrimination. Article 14 states:

“The enjoyment of the rights and freedoms set forth in this Convention shall be secured without discrimination on any ground such as sex, race, colour, language, religion, political or other opinion, national or social origin, association with a national minority, property, birth or other status.”

The Government argued that the refusal of the residency permit was justified on grounds of public health interest. The Court disagreed and held that the applicant had been a victim of discrimination on account of his health status, that being his HIV status. The Court held: “the Court considers that a distinction made on account of one’s health status, including such conditions as HIV infection, should be covered—either as a form of disability or alongside with it—by the term ‘other status’ in the text of Article 14 of the Convention”. Because the Court held that health status, and therefore HIV status, fell under “other status” of Article 14, Article 14 was applicable for consideration in this case. It was concluded that:

“The Court therefore considers that people living with HIV are a vulnerable group with a history of prejudice and stigmatisation and that the state should be afforded only a narrow margin of appreciation in choosing measures that single out this group for differential treatment on the basis of their HIV status.”

The Court ultimately found that there had been a violation of Article 14 of the Convention in conjunction with Article 8 and that My Kiyutin’s application for residency should not have been denied on the basis of his HIV status. The State had to pay damages to Mr Kiyutin.

In making the link between grounds upon which discrimination is prohibited and vulnerability, the Court concluded that persons living with HIV are deserving of protection of a vulnerable group due to the discrimination and stigmatisation they incur. This illustrates the link between recognition of grounds of unfair discrimination and vulnerable groups. 

What this case also illustrates is the greater impacts of recognising persons living with HIV as a vulnerable group. If Mr Kiyutin’s application was denied or his case failed, then he would have been unable to go home with his family, which would necessarily have an impact on his treatment, care, and support. By recognising him as a vulnerable person on the basis of his HIV status, the Court removed barriers to access his necessary treatment, care, and support. The discrimination amounted to an impediment to the realisation of his health rights and also amounted to a violation of the right not to be discriminated against. This case serves as an example of the impact of discrimination on access to treatment, care, and support – albeit it indirectly, as the case did not explicitly concern health rights. The case also shows how recognition of vulnerability, because of the discrimination faced, can offer protection and aid in the realisation of rights.

### 3.2. Advantages of Recognising Persons Living with HIV as Vulnerable 

As argued by Peroni and Timmer [30]: “vulnerability might actually be a useful guiding principle: in the prioritization of scarce resources, states give preference to those whose needs they consider most pressing”. Recognising persons living with HIV as a vulnerable group may remove barriers in accessing their necessary treatment, care, and support. It may also aid in protecting them further from discrimination as clearly contemplated by non-discrimination clauses. 

Persons living with HIV are subject to discrimination and stigmatisation. For this reason, they may be considered vulnerable. This link needs to be made as it can ensure that barriers to realising rights such as healthcare are not impeded by the discrimination faced and also that special measures may be taken to offer protection against discrimination and its subsequent affects. It should not be the case that only persons living with HIV who also belong to another vulnerable group are afforded protection against discrimination and its consequences. Persons living with HIV should be considered vulnerable in and of themselves. If they meet the criteria, under health status or other status, to not be discriminated against, then surely, they meet the criteria of being a vulnerable group. This recognition is arguably necessary to ensure adequate legal protection against unfair discrimination and to ensure that the health rights in terms of treatment, support, and care of such persons are adequately addressed.

## 4. Conclusions

This paper has considered whether persons living with HIV should be considered a vulnerable group by virtue of their recognition not to be discriminated against under human rights law, both internationally and domestically. The case of *Kiyutin v Russia* illustrates how persons living with HIV can and have been recognised as a vulnerable group. The argument is made that on the basis of persons living with HIV being subject to discrimination and stigmatisation, and therefore offered protection under non-discrimination clauses, they should also be considered a vulnerable group. Often it is only persons living with HIV who also belong to a vulnerable group that receive this additional protection, but this paper argues that persons living with HIV should be considered a vulnerable group in and of themselves.

Persons living with HIV are deserving of special measures as they are commonly subject to discrimination, as examined above. Furthermore, the denial of rights on the basis of a person’s HIV status is discriminatory, as contemplated by international human rights law texts, and thus special provision to protect against discriminatory practices is necessary, arguably because this group is vulnerable by virtue of being discriminated against and marginalised by society. As argued by Edge, “only by improving the rights of these people can they have better access to prevention, treatment, and management”.

## Data Availability

Not applicable.
